# Automated assessment of bone changes in cross-sectional micro-CT studies of murine experimental osteoarthritis

**DOI:** 10.1371/journal.pone.0174294

**Published:** 2017-03-23

**Authors:** Patricia Das Neves Borges, Tonia L. Vincent, Massimo Marenzana

**Affiliations:** 1 Department of Bioengineering, Imperial College London, London, United Kingdom; 2 Kennedy Institute of Rheumatology, Nuffield Department of Orthopaedics, Rheumatology and Musculoskeletal Sciences, University of Oxford, Roosevelt Drive, Oxford, United Kingdom; Rush University Medical Center, UNITED STATES

## Abstract

**Objective:**

The degradation of articular cartilage, which characterises osteoarthritis (OA), is usually paired with excessive bone remodelling, including subchondral bone sclerosis, cysts, and osteophyte formation. Experimental models of OA are widely used to investigate pathogenesis, yet few validated methodologies for assessing periarticular bone morphology exist and quantitative measurements are limited by manual segmentation of micro-CT scans. The aim of this work was to chart the temporal changes in periarticular bone in murine OA by novel, automated micro-CT methods.

**Methods:**

OA was induced by destabilisation of the medial meniscus (DMM) in 10-week old male mice and disease assessed cross-sectionally from 1- to 20-weeks post-surgery. A novel approach was developed to automatically segment subchondral bone compartments into plate and trabecular bone in micro-CT scans of tibial epiphyses. Osteophyte volume, as assessed by shape differences using 3D image registration, and by measuring total epiphyseal volume was performed.

**Results:**

Significant linear and volumetric structural modifications in subchondral bone compartments and osteophytes were measured from 4-weeks post-surgery and showed progressive changes at all time points; by 20 weeks, medial subchondral bone plate thickness increased by 160±19.5 μm and the medial osteophyte grew by 0.124±0.028 μm^3^. Excellent agreement was found when automated measurements were compared with manual assessments.

**Conclusion:**

Our automated methods for assessing bone changes in murine periarticular bone are rapid, quantitative, and highly accurate, and promise to be a useful tool in future preclinical studies of OA progression and treatment. The current approaches were developed specifically for cross-sectional micro-CT studies but could be applied to longitudinal studies.

## Introduction

Osteoarthritis (OA) is the most prevalent joint disease; an incurable and painful condition that is a leading cause of disability worldwide. Although cartilage degradation is a hallmark of disease, OA is regarded as an ‘organ failure’, involving the whole joint [1, 2]. Many changes occur in bone, including attrition, sclerosis, formation of osteophytes, cysts, and marrow lesions [1, 3]. Excessive bone remodelling has been linked to cartilage degeneration [4, 5] and pain [6] from early on in disease [7], but the nature of the relationship between both tissues and how lesions progress over time remains unclear [8, 9]. This is partly because cartilage loss frequently progresses prior to development of symptoms and partly because available tools are insensitive and do not permit early diagnosis [10]. In clinic, disease progression is mostly assessed by radiographic scoring using semi-quantitative systems [11–13] once bone changes are well-established, but this evaluation lacks the sensitivity to track temporal changes [14]. Furthermore, tissue is usually only available at the late stages of disease, keeping early events poorly understood. Therefore, experimental models hold a key role, not only to help understand pathogenesis and to chart temporal changes in bone and cartilage, but also to develop strategies for early detection and therapeutic targeting [10]. The mouse model, largely due to its being amenable to genetic modifications, is widely used in research. In the recent years, micro-computed tomography (micro-CT) has become the gold-standard imaging modality for bone assessment in this model, owing to excellent resolution, 3D capability, and utility in longitudinal studies [15]. However, micro-CT lacks validated methodologies for automated analysis of the epiphyseal subchondral bone, and mostly for structure segmentation, which is frequently based on manual contouring of regions-of-interest [16–18]. To increase segmentation throughput, automated approaches have been proposed; mostly for compartmentalisation of cortical and trabecular bone [19–22]. While useful, these methods often rely on thresholds, based on the premise that cortical and trabecular bone can be differentiated by their different grey level intensities. The choice of an appropriate threshold is, however, critical for accurate segmentation and minor changes may cause errors [22], leading to mis-estimation of structural parameters [15]. Accuracy on compartmentalisation can be improved by combining thresholding methods and microstructural criteria, such as thickness differences between cortical and trabecular bone [23, 24]. Additionally, despite osteophytes being a well-established feature of osteoarthritic joints, there seems to be a lack of validated methods for measuring these bony structures. Assessment in both clinical [12] and experimental models [25] has been often limited to semi-quantitative grading based on their size and maturity, but micro-CT scans have been shown to have the potential to provide volumetric measurements of osteophytes [26].

There is a need for sensitive, high-throughput, quantitative methodologies that can be applied in experimental OA to chart bone changes in disease. In this work, we describe a novel set of image analysis methods based on 3D image registration of micro-CT datasets, acquired using an *ex vivo* scanner, that allows bony structures in mouse tibial epiphyses to be automatically compartmentalised and quantified, improving the speed, quantitation, and reproducibility of existing measurements. In the present cross-sectional study, we chart the structural modifications in bone over 20 weeks following surgical joint destabilisation. We validated our findings against manual segmentation as well as histopathology, and showed that the proposed compartmentalisation method can be applied to datasets obtained from rescanning our samples using an *in vivo* micro-CT scanner (with lower resolution), which simulate datasets generated in longitudinal studies.

## Materials and methods

### Animals and surgical destabilisation of the medial meniscus

Animal work was approved by the Home Office and conducted according to the Animals (Scientific Procedures) Act 1986. Male C57Bl/6 mice (seven groups, n = 6, 10-week old) were purchased from Charles River (UK Ltd, Margate, UK). Six groups underwent surgical destabilization of the medial meniscus (DMM) on the right knee [27], while the left joint was used as a control (contralateral). Mice were euthanized 1-, 2-, 4-, 8-, 12- and 20-weeks post-surgery. One group of animals was used as a non-operated healthy baseline and was sacrificed at the time of surgery (indicated as time 0).

### Micro-computed tomography imaging

Joints were disarticulated, tibiae dissected and re-hydrated in saline. Proximal tibiae were imaged in a micro-CT scanner (SkyScan1172 X-ray microtomograph, Antwerp, Belgium) within saline (5 μm/pixel, 50 kV, 200 μA, 1600 ms exposure, 2 frames/projection, 0.6° angular step, 180° scan, 5.0×4.5 mm field of view, 27 minutes of acquisition time). Tomograms were reconstructed in NRecon software (V1.6.5.2, SkyScan, Antwerp, Belgium) using an algorithm that included ring artefact reduction, beam-hardening correction, and misalignment compensation. For calibration of bone mineral density (BMD), high and low density phantoms (0.75 and 0.25 g/cm^3^ of calcium hydroxyapatite, respectively) were imaged using the same settings of tibiae. Following reconstruction, the average grey level intensity within a volume-of-interest was measured in both scans and a linear calibration was derived between the grey level intensity and BMD.

### Surface heat maps of subchondral bone thickness

To visualise and localise changes in subchondral bone, we generated planar heat maps of thickness on the top surface of tibial epiphyses and extracted profiles. Micro-CT coronal views were exported to ImageJ (US National Institutes of Health, Bethesda, Maryland, USA) to calculate the volumetric thickness [28]. Using Matlab (R2011b, The MathWorks Inc., Natick, MA, USA), colour-coded maps were generated by projecting the thickness of the epiphyseal top edge onto a plane. To directly estimate the amplitude of the changes, but not for quantitative purposes (which are served by the volumetric analysis described below), profiles were computed along the medial-lateral planes in fixed-size regions (750 μm in height) that included most of the load-bearing regions and according to a methodology we previously described [29].

### 3D image registration

**Fig 1:**
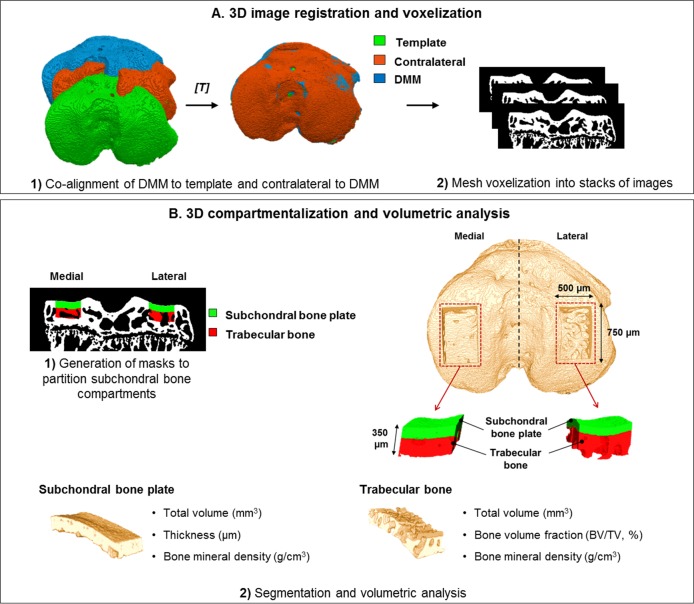
Flowchart of the automated image analysis method for subchondral bone partition into plate and trabecular bone and volumetric quantifications. In the first stage (A), 3D image registration was applied to micro-CT scans of DMM tibiae for alignment to a template position, while the contralateral was co-aligned with the correspondent ipsilateral (step 1 in A). Prior compartmentalisation and volumetric analysis (B), meshes were voxelized into stacks of images (step 2 in A). Volume partition was performed based on macro-porosity differences, generating two colour-coded volumes-of-interest for each aspect of the tibial plateau (medial and lateral), green for subchondral bone plate and red for trabecular bone (step 1 in B). Using these mappings, the original volumes-of-interest were extracted for quantitative analysis of each compartment (step 2 in B). The top view of the 3D model of the tibial epiphysis shows the dimensions and location of the mappings across the load-bearing areas and the dashed line demarcates the medial and lateral aspects of the plateau.

To ensure consistency in the volumetric analysis of subchondral bone and to allow for subsequent shape comparisons between pairs of tibiae, we applied 3D image registration to the micro-CT scans (Fig 1A). In this approach, the DMM tibia was aligned to a template position, used to correct for the inherent differences in specimen orientation during imaging, while the contralateral was co-aligned to the corresponding ipsilateral (step 1 in Fig 1A). In this way, the subsequent selection of volumes-of-interest for analysis was not only consistent between pairs of tibiae, but also among animals. Scans were binarized by means of global thresholding calculated using Otsu’s method [30] by which the value of 60 (in a scale ranging between 0 and 255) was found to be optimal. Meshes were generated (CTAnalyzer, V1.13.5.1, Skyscan, Antwerp, Belgium) and fed into the Image Registration Toolkit (IRTK, Ixico Ltd., UK), which is fully documented at https://biomedia.doc.ic.ac.uk/software/irtk/. This open-source toolkit is composed of libraries and command-line tools for image processing and analysis (https://www.doc.ic.ac.uk/~dr/software/usage.html). Different types of registration can be performed, including rigid surface registration between two input meshes (https://www.doc.ic.ac.uk/~dr/software/usage.html#srreg). Briefly, this tool estimates, for each point in the target mesh, the closest location in the source mesh, resulting in two sets of corresponding points. In an iterative process, it then calculates the least squares fit for the distance between corresponding points, and estimates a matrix of transformation, *T* [31]. The estimated transformation is then applied to transform the target mesh, using the surface transformation tool (https://www.doc.ic.ac.uk/~dr/software/usage.html#transformation), which utilizes the nearest neighbour interpolation method to produce a registered mesh aligned to the source. Meshes outputted by the registration tools were voxelized from stereolithography (.stl) format in Matlab (www.mathworks.com/matlabcentral/fileexchange/27390-mesh-voxelisation), resulting in stacks of registered images (in bitmap format) whose resolution was the same as the original scans (step 2 in Fig 1A).

### Automated analysis of subchondral bone

We performed analysis of individual subchondral bone compartments using a bespoke method developed in Matlab that compartmentalises and quantifies epiphyseal bone (Fig 1B). Upon binarization using Otsu’s method [30], morphological image processing (opening and closing operations) using a disk-shaped kernel was applied to remove any remaining noise from thresholding. For volume partitioning into plate and trabecular bone, the middle coronal plane was selected, the proximal edge of the tibial plate surface detected, and its medial and lateral extremes determined. The centre of each aspect of the plateau was estimated based on fixed widths from these two extremes and expanded by 500 μm. Following the calculation of mapping locations, two fixed-size regions (500 μm in width by 350 μm in height) were placed by line drawing in each portion of the plateau. Subchondral bone was subdivided into plate and trabecular bone based on macro-porosity; this criterion was chosen based on the knowledge of bone macrostructure which is either cortical, a compact structure with low porosity, or trabecular, a lattice-type and highly porous structure [32]. Furthermore, the degree of bone porosity is commonly used as visual criterion to segment CT data. Our algorithm determined line-wise, and through the 350 μm of depth, the proportion of foreground (bone) to background (pores) pixels, which gives a measurement of bone volume fraction. The transition between compartments occurred when the measurement of bone volume fraction was less than 90% and a different colour-coding was assigned to each compartment: green for the plate (macro-porosity < 10%) and red for trabecular bone (macro-porosity ≥ 10%). To constrain the volumes-of-interest to the tibial load-bearing areas, mappings were extended over 750 μm along the anterior-posterior axis (375 μm anterior and 375 μm posterior from the middle coronal plane initially selected) (illustrated in the 3D model shown in Fig 1B). After mapping, the volumes enclosed by the masks were segmented and volumetric parameters calculated. For the subchondral bone plate, the total volume of the compartment, thickness, and BMD were measured. For trabecular bone, the total volume of the compartment, bone volume fraction, measured as the ratio between the bone content and the total volume of the compartment (BV/TV, expressed in percentage), and BMD were calculated (step 2 in Fig 1B).

### Validations

We validated our method by comparing measurements in automated and manually-drawn volumes-of-interest. In the scans from non-operated baseline animals (10-week old, n = 6), compartments in both right and left tibiae were manually segmented into subchondral bone plate and trabecular bone (CTAnalyzer). The same extend across the anterior-posterior planes was analysed and microstructural parameters (subchondral bone plate volume and thickness, and trabecular total volume, bone volume, and BV/TV) were measured using the routines from the automated methodology.

Furthermore, in our cross-sectional study, we have analysed subchondral bone compartments with and without pre-alignment by 3D rigid registration to compare the variability of measurements in both alignment conditions.

### Applicability to lower resolution scans to simulate datasets from longitudinal studies

To test the applicability of the compartmentalisation method to lower resolution scans, such as the ones obtained from *in vivo* scanners, a group of undissected hind limbs from cadaveric mice (male C57Bl/6, 10-week old, n = 5) was scanned using an *in vivo* scanner (Quantum FX, PerkinElmer, USA). Scans were acquired at an isotropic voxel size of 10 μm (70 kV, 160 μA, 5.0×5.0 mm field of view, 3 minutes of acquisition time) and reconstructed using the manufacturer built-in software. Subsequently, tibiae were finely dissected and re-imaged in the *ex vivo* scanner using the methodology described above. We tuned the bespoke compartmentalisation method to accommodate for the pixel resolution of the *in vivo* scanner (increasing from a pixel size of 5 to 10 μm); extension of the mappings and criterion for subchondral bone compartmentalisation were kept unaltered.

### Osteophyte quantification using 3D image registration

To identify and quantify medial osteophytes, we implemented a method in Matlab based on 3D image registration. Upon co-alignment of DMM/contralateral pairs of tibiae using the methodology described above, binarized stacks of images underwent image processing. This included morphological operations (closing and dilation) using a disk-shaped kernel followed by cavity filling. Whole structure filling was justified by prior knowledge of the DMM model in which osteophytes are observed as an outgrowing protrusion. Internal differences in bone structure are not relevant in this assessment; only size and shape differences between healthy contralateral and operated tibiae. To determine the volumetric differences between pairs of tibiae, image subtraction was applied. Medial differences were subsequently enclosed in a fixed-size volume-of-interest and quantified as a volumetric shape difference caused by the osteophyte. To help visualising shape differences, we colour-coded the DMM tibiae in red and the correspondent contralateral in green, and therefore, the overlap between structures would be coloured yellow, except for any structure that differed from the healthy shape. For validation, we manually segmented osteophytes from micro-CT coronal views (CTAnalyzer) and calculated their volume upon cavity filling.

### Osteophyte quantification using epiphyseal volume

We used whole epiphyseal volume as a surrogate measurement of the expansion caused by osteophytes. To do so, tibial epiphyses were manually segmented from micro-CT coronal views (CTAnalyzer) and following image processing procedures, which included Gaussian filtering (σ = 2), morphological operations (closing and dilation) using a disk-shaped kernel, and cavity filling, the total volume was quantified.

### Histopathology

For articular cartilage scoring, tibiae were decalcified, dehydrated, and coronally embedded in paraffin. Sections were taken at regular spacing across the samples (80 μm between each level in a total of twelve levels) and stained using Safranin-O. Medial and lateral aspects of the tibial plateau were subsequently graded depending on the degree of cartilage damage according to a scoring system previously detailed [33, 34] that was modified from the semi-quantitative system originally defined by Glasson *et al*. [35]. The final score for each aspect of the plateau was obtained from the sum of the scores of all levels.

### Statistical analysis

Statistics were computed using GraphPad Prism 7.02 (San Diego, CA, USA). Data was expressed as mean ± 95% confidence intervals and verified for normality using Shapiro-Wilk tests. For the cross-sectional study, two-way analysis of variance (ANOVA) followed by *post hoc* multiple comparison tests using Bonferroni correction were used to determine statistical differences among groups. The effect of time on bone changes within each compartment was determined by comparisons between the measurements in healthy baseline mice (time 0) with the subsequent time points upon surgery (represented in the graphs by the symbol *). The effect of the DMM surgery was determined by comparisons between operated *vs*. contralateral tibiae at each time point (represented in the graphs by the symbol #). The same approach was used to study differences in whole epiphyseal volume over time. The percentage difference of contralateral epiphyseal volume was tested by one-way ANOVA followed by multiple comparison tests using Bonferroni correction to determine differences between measurements in healthy baseline and subsequent time points. The same statistical tests were applied to study differences in automated osteophyte measurements obtained by 3D registration. Two-way ANOVA followed by multiple comparison tests using Bonferroni correction was applied to determine statistical differences between automated and manual measurements of osteophyte volume from 2-weeks post-DMM. Since histopathology scores of articular cartilage did not follow normal distributions, non-parametric analyses by Kruskal-Wallis tests followed by Dunn’s multiple comparison tests were applied to determine statistical differences between scores at 1-week post-DMM and the subsequent time points for each aspect of the plateau.

To validate our automated methods, we determined the parametric correlation (Pearson correlation coefficient, *r*) between the measurements obtained by manual and automated segmentation, assessed long-term precision error by calculating the root mean square (RMS) error from the residuals of the linear regression, and the precision error of repeated measurements by the coefficient of variation (CV). The CV was calculated as the ratio between the standard deviation to the mean of the measurements, and expressed in percentage [36].

The agreement between automated and manual segmentation of subchondral bone compartments and osteophyte was studied by Bland-Altman plots, where the difference between the measurements was plotted against the average, according to the original graphical approach [36]. The mean, expressing the bias of the measurements, and limits of agreement for each microstructural parameter are reported. Linear regressions of the differences were further plotted to investigate proportional differences between the methods [37].

Non-parametric correlation analysis (Spearman correlation coefficient, *r’*) was used to study the association among lesions in subchondral bone, osteophyte formation and articular cartilage degradation. Values were considered statistically different at *P<0.05, **P<0.01, ***P<0.001 and ****P<0.0001 (symbols * and ^#^ represent the same level of significance).

## Results

### Automated subchondral bone compartmentalisation is accurate and reproducible

We validated our method by comparing quantifications using automated mappings and manual segmentation in non-operated baseline animals (Table 1). Using parametric correlation analysis, we found good agreement between measurements using both methods, thus indicating correct and reproducible subchondral bone compartmentalisation into plate and trabecular bone. Significant correlations were found for all microstructural parameters except for the medial trabecular total volume (p = 0.088), despite the moderate correlation coefficient. The RMS errors, obtained from linear regression analysis, indicated that the automated method has good accuracy; in subchondral bone plate volume measurements, we found that errors were limited to 2.6% and 4.2% of the average values (measured automatically) for medial and lateral aspects of the plateau, respectively, while in the thickness measurements these were 5.6% and 2.4% of the averages. In the trabecular compartment, RMS errors were also limited to small percentages of the averages; in total volume, we found errors of 4.0% and 3.1% in the medial and lateral aspects, respectively, 6.1% and 4.1% in the bone volume, and 6.0% and 3.0% in the BV/TV measurements. In general, the %CV in automated measurements was lower than the one obtained by manual segmentation, although in both cases variation levels were very similar (average %CV of 7.7% using both segmentation methodologies).

**Table 1:** Mean and standard deviation (SD), Pearson correlation coefficients, root mean square (RMS) errors and coefficients of variation (%CV) of the measurements in manually and automatically segmented subchondral bone compartments.

Microstructural parameter		Mean ± SD		Pearson *r*	RMS error	Coefficient of Variation (%)	
		Automated	Manual			Automated	Manual
Subchondral bone plate volume (mm^3^)	Medial	0.0546±0.0072	0.0564±0.0055	0.97****	0.0014	13.2	9.8
	Lateral	0.0431±0.0043	0.0440±0.0040	0.92****	0.0018	10.0	10.0
Subchondral bone plate thickness (μm)	Medial	143.3±11.8	138.9±12.2	0.78**	8.0	8.3	8.8
	Lateral	112.9±7.9	110.9±9.3	0.92****	2.7	7.0	8.4
Trabecular total volume (mm^3^)	Medial	0.0744±0.0062	0.0728±0.0033	0.51	0.0030	8.4	4.5
	Lateral	0.0871±0.0039	0.0843±0.0041	0.78**	0.0027	4.4	4.9
Trabecular bone volume (mm^3)^	Medial	0.0495±0.0030	0.0486±0.0044	0.76**	0.0030	6.1	9.0
	Lateral	0.0420±0.0019	0.0410±0.0024	0.73**	0.0017	4.6	5.9
Trabecular BV/TV (%)	Medial	67.0±5.5	66.7±5.8	0.78**	3.8	8.2	8.7
	Lateral	48.3±3.1	48.7±3.2	0.91****	1.4	6.5	6.6

Subchondral bone plate volume, thickness, and trabecular total volume, bone volume, and BV/TV measured in the medial and lateral aspects of the tibial plateau upon automated and manual compartmentalisation were paired and analysed. The mean and standard deviation (SD) of each parameter is reported as well as the Pearson’s correlation coefficient (*r)* obtained from parametric analysis (**P<0.01 and ****P<0.0001). The RMS error obtained from linear regression analysis and the %CV in both cases are also reported (n = 12 in which medial/lateral aspects of the plateau in right and left legs were pooled together).

**Fig 2:**
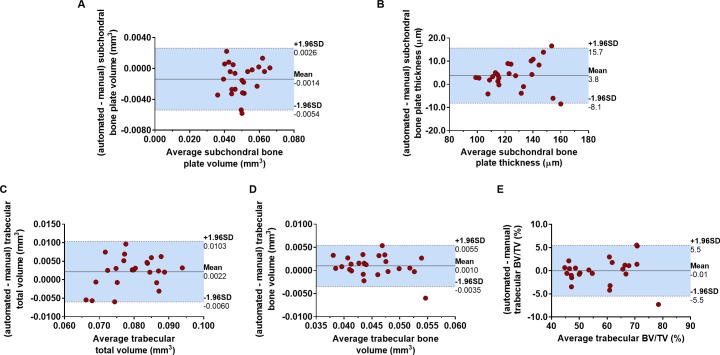
Bland-Altman plots to determine the agreement between the measurements in automatically and manually segmented subchondral bone compartments. The difference between measurements using automated and manual segmentation was plotted against the average for the subchondral bone plate (A) volume and (B) thickness, and trabecular (C) total volume, (D) bone volume and (E) BV/TV, (n = 24 in which the measurements in the medial and lateral aspects of the plateau for both right and left legs were pooled together). The mean (solid line) and the 95% limits of agreement (-1.96SD, +1.96SD, indicated by the area shaded in blue between the dashed lines) for each microstructural parameter are reported in the plots.

Fig 2 displays Bland-Altman analyses, where the difference between automatically and manually obtained measurements were plotted against the averages. In general, good agreement was found between methodologies for all microstructural parameters. In subchondral bone plate volume, the bias was -0.0014 mm^3^, while in the thickness, the bias was 3.8 μm (2.8% and 3.0% of the average values, respectively). These biases had a trend to increase with the measured average value, but the coefficient of determination was limited to r^2^ = 0.018 (p = 0.54) for the volume measurement, and r^2^ = 0.018 (p = 0.53) for the thickness measurement, which indicated a negligible proportional relationship within the measurement range of interest. In trabecular bone, the bias in total volume measurement was 0.0022 mm^3^, 0.0010 mm^3^ in bone volume (2.7% and 2.2% of the average values, respectively) and -0.01% in BV/TV, which we can consider negligible. The bone volume bias had a trend to decrease with the measured average value, while the total volume had a trend to increase with the average. Also in these measurements, the coefficients of determination of the differences, r^2^ = 0.070 (p = 0.21) for the bone volume, and r^2^ = 0.103 (p = 0.13) for the total volume, indicated negligible proportional relationships within the ranges of interest of the measurements.

Furthermore, pre-alignment of the micro-CT scans using 3D image registration reduced the %CV of the automated measurements in subchondral bone compartments (S1 Table), thus indicating that its application increased method robustness.

### Heat maps of subchondral bone thickness can distinguish DMM and contralateral tibiae over time from 2-weeks post-surgery

**Fig 3:**
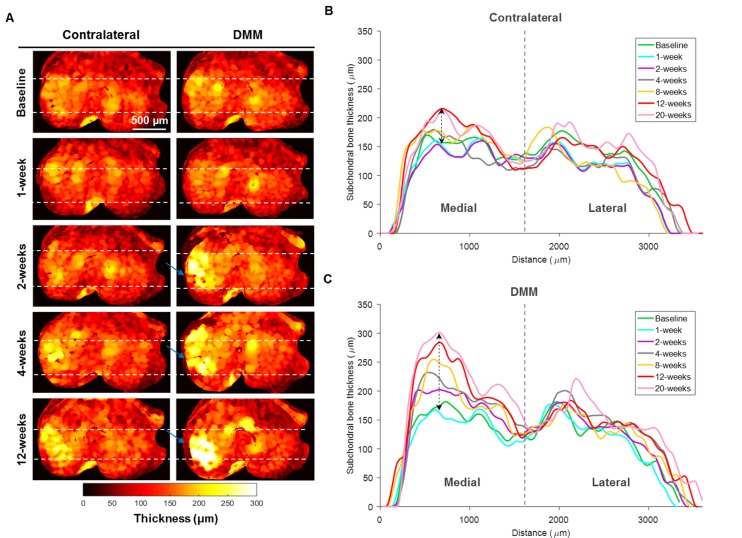
Heat maps and profiles of subchondral bone thickness obtained by micro-CT over OA temporal progression. (A) Representative pairs of contralateral (left) and DMM-operated (right) tibial top surfaces at baseline, 1-, 2-, 4- and 12-weeks post-DMM, showing progressive subchondral bone thickening in the medial compartment of DMM tibiae, where colour-coding changed from orange (~150 μm) at 1-week towards white (~300 μm) at 12-weeks. Blue arrows in DMM maps indicate the presence of medial osteophytes and dashed white lines delimit the location where medial to lateral profiles were extracted for both contralateral (B) and DMM-operated (C) tibiae. In destabilised joints (C), the peak thickness in the medial compartment was continuously increased from baseline to 20-weeks post-DMM (increment of ~135 μm, indicated by the dashed arrow) while in contralateral (B) this difference was less marked (~50 μm, indicated by the dashed arrow). The superimposition of all profiles in the lateral compartment suggested that no changes occurred in this area.

Fig 3A shows representative surface heat maps of subchondral bone thickness from the tibiae of DMM-operated and contralateral joints in the weeks following surgery. Fig 3B and 3C display thickness profiles from medial to lateral planes through the load bearing areas of tibiae. At healthy baseline and 1-week post-surgery, no differences between contralateral (left) and DMM (right) tibiae were found; maps and profiles were similar with maximum values of thickness of ~175 μm located in the centre of the medial and lateral aspects of the plateau. However, from 2-weeks post-surgery, medial subchondral bone thickening in destabilised joints became apparent (colour-coding changing from orange, corresponding to a thickness ranging between ~150–175 μm, towards white, corresponding to a thickness ranging between ~275–300 μm). Greatest changes were seen in the load-bearing area of the medial compartment, and the lateral compartment was relatively spared. Interestingly, from 4-weeks onwards, thickening of subchondral bone within the medial compartment in the contralateral joint was also visible (~50 μm, indicated by dashed arrow in Fig 3B), although this was less marked than in the destabilised joint (~135 μm, indicated by dashed arrow in Fig 3C). Osteophytes were evident at the medial margin of destabilised joints (blue arrows in Fig 3A); the newly formed structure was poorly ossified at 2-weeks and had low thickness (~30 μm), whereas at later stages was ossified (~75 μm) and extended along the epiphyseal margin. Furthermore, the thickness peak in DMM-operated appeared to shift laterally (towards the right side of Fig 3C) as the medial osteophyte increased its volume.

### Subchondral bone sclerosis replaces trabecular bone in the medial compartment following DMM surgery

**Fig 4:**
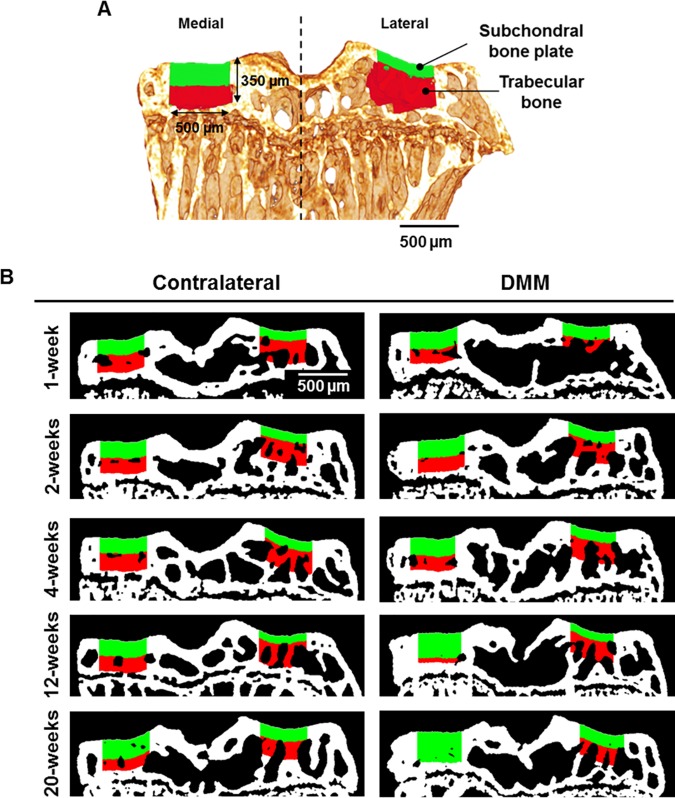
Automated compartmentalisation of epiphyseal subchondral bone over OA temporal progression. (A) Middle coronal view of a 3D model showing epiphyseal compartmentalisation into subchondral bone plate (colour-coded in green) and trabecular bone (colour-coded in red) in the medial and lateral aspects of the tibial plateau, demarcated by the dashed line. (B) Representative coronal views of the mappings (500 μm in width by 350 μm in depth) at 1-, 2-, 4-, 12- and 20-weeks post-DMM, for both contralateral and DMM tibiae.

Our method consistently partitioned epiphyseal subchondral bone into plate and trabecular bone compartments (Fig 4A) and allowed automated and high-throughput screening of subchondral bone changes. We applied it across all the experimental groups at different time points post-DMM (Fig 4B) and mappings showed plate thickening in medial DMM tibiae. This appeared to involve a progressive enlargement of the cortical compartment (colour-coded in green) at the expenses of the trabecular compartment (colour-coded in red). The lateral side remained unaltered, an observation consistent with thickness maps and profiles (Fig 3).

**Fig 5:**
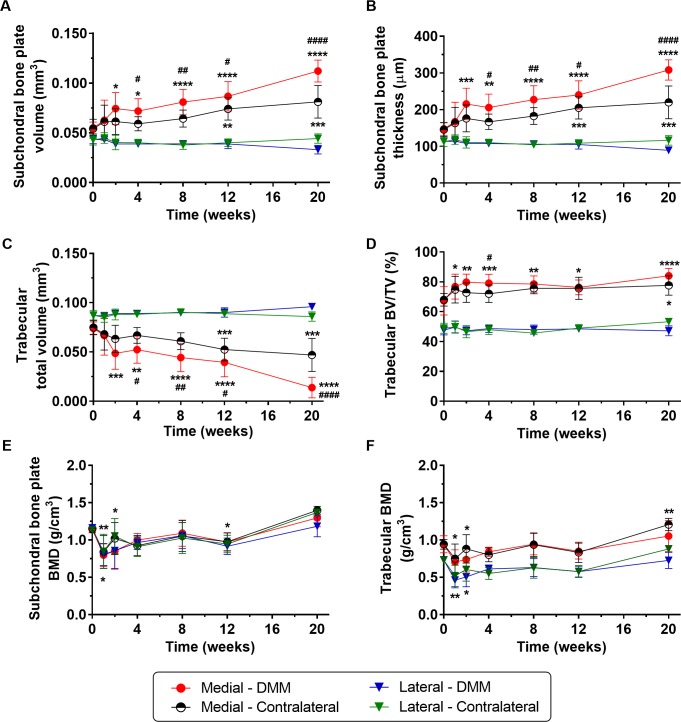
Microstructural assessment of subchondral bone compartments. Subchondral bone plate (A) volume and (B) thickness, and trabecular (C) total volume and (D) BV/TV, (E) subchondral bone plate BMD, and (F) trabecular BMD within the automated volumes-of-interest of medial and lateral aspects of the plateau for DMM-operated and contralateral tibiae (n = 6, * denotes comparisons between measurements in the non-operated baseline (time 0) with subsequent time points post-surgery, while # denotes comparisons between DMM-operated *vs*. contralateral aspects of tibial plateau at the same time point, obtained by two-way ANOVA followed by *post hoc* multiple comparison tests using Bonferroni correction). *P<0.05, **P<0.01, ***P<0.001 and ****P<0.0001 (both * and ^#^ symbols denote the same levels of significance).

We found medial plate sclerosis in DMM tibiae compared to contralateral as early as 4-weeks post-surgery. Increased volume (p = 0.031, Fig 5A) and thickness (p = 0.012, Fig 5B) were observed and measurements continued to increase up to 20-weeks. Medial DMM measurements were also significantly elevated from healthy baseline (0-weeks) as early as 2-weeks post-surgery (p = 0.016 for plate volume, Fig 5A, and p = 0.0007 for plate thickness, Fig 5B). On average, we observed an increase of 160 μm in plate thickness, from baseline to 20-weeks, p<0.0001, which was in good agreement with the thickness profiles (dashed arrow in Fig 3C). A dynamic response was evident in the medial contralateral joint (as indicated by the thickness profiles, Fig 3B) with significant plate thickening (average increase of 74 μm, p = 0.004, for baseline vs. 20-weeks, Fig 5B). No changes were observed on the lateral side of DMM-operated and measurements remained steady (average measurements of 0.040 mm^3^ of volume and 110 μm of thickness, p = 0.77 and p>0.99, for 0- vs. 20-weeks measurements of volume and thickness, respectively, Fig 5A and 5B). Due to medial plate sclerosis in destabilised joints, the cortical compartment expanded (average increment of 0.057 mm^3^ in volume from 0- to 20-weeks, p<0.0001, Fig 5A) leading to an opposite behaviour in the trabecular bone compartment, whose volume decreased from 0.074 (0.067, 0.081) mm^3^ at baseline to 0.014 (0.003, 0.024) mm^3^ at 20-weeks post-surgery (Fig 5C). We also found elevated trabecular BV/TV in the medial compartment at 4-weeks post-DMM compared with contralateral (p = 0.02) and at 1-week post-DMM compared with baseline (p = 0.03), while lateral measurements remained unaltered (Fig 5D). Despite plate sclerosis (Fig 5A and 5B) and trabecular bone remodelling (Fig 5D), we found no differences in BMD between operated and non-operated joints in either of the compartments (Fig 5E and 5F); these were only noted within compartments at early and late time points of disease.

### Methodology is applicable to lower resolution scans

To evaluate the applicability of our methodology in lower resolution scans and, potentially in longitudinal studies, we scanned samples using an *in vivo* micro-CT scanner (which has lower resolution compared to the *ex vivo* scanner) and evaluated how the accuracy of our automated analysis was affected by these lower resolution datasets compared with our standard higher resolution datasets (Table 2). Overall, we found that the segmentation of subchondral bone compartments was highly correlated irrespective of resolution despite overestimating volumes and lengths in the lower resolution datasets (data in the supporting information S1 Dataset). Decreased resolution mostly affected trabecular bone measurements, which did not correlate well for both medial and lateral bone volume and lateral BV/TV (p = 0.25, p = 0.59, and p = 0.88, respectively). This is, however, an expected effected due to the small dimensions of trabeculae in mouse bone, which are more prone to errors in structural measurements using lower resolution scans. Furthermore, most of the %CV increased with the increment in voxel size (average %CV of 16.0% at 5 μm/pixel and 22.8% at 10 μm/pixel resolution). The RMS errors in subchondral bone plate volume measurements were 11.6% and 7.8% of the average values (measured at 10 μm/pixel resolution) for medial and lateral aspects of the plateau, respectively, while in the thickness measurements these were 12.5% and 8.6% of the averages. In the trabecular compartment, RMS errors in total volume were 20.4% and 3.6% in the medial and lateral aspects, respectively, 14.7% and 4.8% in the bone volume, and 5.6% and 7.3% in BV/TV measurements.

**Table 2:** Mean and standard deviation (SD), Pearson correlation coefficients, root mean square (RMS) errors and coefficients of variation (%CV) of the measurements in subchondral bone compartments imaged at 5 and 10 μm/pixel resolution.

Microstructural parameter		Mean ± SD		Pearson *r*	RMS error	CV (%)	
		5 μm/pixel	10 μm/pixel			5 μm/pixel	10 μm/pixel
Subchondral bone plate volume (mm^3^)	Medial	0.0606±0.0170	0.0825±0.0259	0.85**	0.0096	28.1	33.5
	Lateral	0.0352±0.0051	0.0412±0.0051	0.81**	0.0032	14.6	16.2
Subchondral bone plate thickness (μm)	Medial	161.6±46.1	210.7±68.9	0.84**	26.3	28.5	32.0
	Lateral	91.3±13.9	100.5±13.7	0.81**	8.6	15.2	17.4
Trabecular total volume (mm^3^)	Medial	0.0688±0.0172	0.0485±0.0258	0.84**	0.0099	25.1	53.3
	Lateral	0.0952±0.0052	0.0898±0.0051	0.81**	0.0032	5.5	5.7
Trabecular bone volume (mm^3^)	Medial	0.0505±0.0060	0.0394±0.0167	0.40	0.0058	11.9	42.5
	Lateral	0.0505±0.0027	0.0585±0.0039	-0.19	0.0028	5.3	6.7
Trabecular BV/TV (%)	Medial	76.2±12.9	85.1±9.2	0.94****	4.8	17.0	9.6
	Lateral	53.3±4.5	65.3±5.0	0.05	4.8	8.5	11.6

Subchondral bone plate volume, thickness, and trabecular total volume, bone volume, and BV/TV of the medial and lateral aspects of the tibial plateau quantified in 5 and 10 μm/pixel scans were paired and analysed. The mean and standard deviation (SD) of each parameter is reported as well as the Pearson’s correlation coefficient (*r)* obtained from parametric analysis (**P<0.01 and ****P<0.0001). The RMS error obtained from linear regression analysis and the %CV for both resolutions are also reported (n = 10 in which medial/lateral aspects of the plateau in right and left legs were pooled together).

### Shape comparisons based on 3D registration enable osteophyte quantification

**Fig 6:**
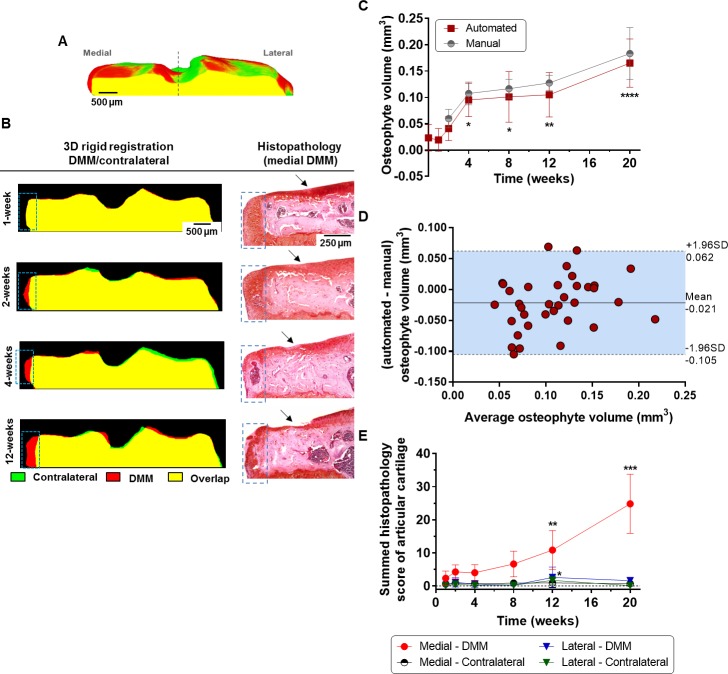
Osteophyte quantification using shape comparisons based on 3D image registration. (A) Middle coronal view of a 3D model showing registration of DMM (colour-coded in red) to contralateral (colour-coded in green) tibia leading to superimposition between structures (colour-coded in yellow). The dashed line demarcates the medial and lateral aspects of the plateau. (B) Representative coronal views of registered DMM/contralateral tibiae showing osteophytes as a medial outgrowing protrusion (highlighted by dashed boxes), which were well validated by histopathology. Progressive articular cartilage damage is indicated by arrows. (C) Osteophyte volume measure either by automated or manual segmentation (n = 5, *P<0.05, **P<0.01 and ***P<0.001, computed by one-way ANOVA followed by multiple comparison tests using Bonferroni correction to determine differences between measurements in healthy baseline and subsequent time points post-surgery). (D) Bland-Altman plot to determine the agreement between the measurements in automatically and manually segmented osteophytes. The difference between measurements using automated and manual segmentation was plotted against the average (n = 35, with the measurements of all time points upon DMM pooled together). The mean (solid line) and the 95% limits of agreement (-1.96SD, +1.96SD, indicated by the area shaded in blue between the dashed lines) are reported in the plot. (E) Articular cartilage summed histopathology scores in medial and lateral aspects of the plateau for DMM and contralateral tibiae (n = 5, **P<0.01 and ***P<0.001 by non-parametric Kruskal-Wallis tests followed by *post hoc* Dunn’s multiple comparisons tests to determine statistical differences between scores at 1-week post-DMM and the subsequent time points for each aspect of the plateau).

We used paired shape comparisons between DMM and contralateral tibiae to automatically segment and quantify osteophytes (Fig 6A). Medial osteophytes in DMM-operated were consistently found after 2-weeks using this method (Fig 6B); while at 1-week post-DMM, they were imperceptible. Quantifications showed two phases of growth (Fig 6C); at the early stages (1- to 4-weeks) where volume increment was rapid (0.019 (-0.002, 0.041) mm^3^ at 1-week to 0.095 (0.063, 0.127) mm^3^ at 4-weeks, p = 0.02) and a second slower at later stages (0.101 (0.053, 0.149) mm^3^ at 8-weeks to 0.165 (0.120, 0.211) mm^3^ at 20-weeks, p = 0.15). We validated the automated measurements using manual segmentation (line plot in grey, Fig 6C) and excellent agreement was found between the two methodologies. However, the %CV of automated measurements (28.3%) was found to be higher than the one in manual measurements (14.2%); the average RMS error was found to be 0.024 mm^3^. Bland-Altman analysis confirmed the good agreement between segmentation methodologies (Fig 6D). We found a bias of -0.021 mm^3^ (17.8% of the average value) in osteophyte measurement and this had a trend to increase with the measured average value. Nevertheless, the coefficient of determination associated with the difference (r^2^ = 0.075, p = 0.11) indicated a negligible proportional relationship between variables.

### Epiphyseal volume provides a surrogate measurement of osteophyte formation

**Fig 7:**
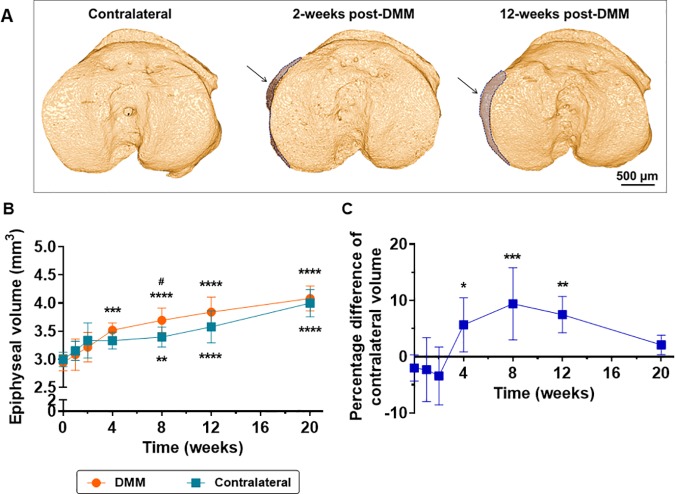
Osteophyte quantification using epiphyseal volume. (A) Top views of tibial epiphyses showing medial expansion caused by osteophytes in DMM-operated (arrows and shaded regions-of-interest) compared with an unaltered contralateral (12-weeks post-surgery). At 2-weeks, the osteophyte appeared incomplete, while at 12-weeks broaden the articular surface. (B) Epiphyseal volume over time for DMM and contralateral tibiae (n = 5, * denotes comparisons between measurements in the non-operated baseline with subsequent time points post-surgery while # denotes comparisons between DMM-operated *vs*. contralateral tibiae at the same time point, obtained by two-way ANOVA followed by multiple comparison tests using Bonferroni correction). ^#^P<0.05, **P<0.01 and ****P<0.0001. (C) Difference between epiphyseal volume of DMM-operated and contralateral tibiae over time expressed as a percentage difference (n = 5, *P<0.05, **P<0.01 and ***P<0.001, computed by one-way ANOVA followed by multiple comparison tests using Bonferroni correction to determine statistical differences between measurements in healthy baseline and subsequent time points).

We asked whether whole epiphyseal volume could be used as a surrogate measurement to assess osteophyte volume. Representative top views of 2- and 12-weeks post-DMM epiphyses show the expansion (highlighted by arrows and shaded regions-of-interest, Fig 7A) due to new bone formation on the medial border of the plateau, whereas the non-operated contralateral (12-weeks post-surgery) was unaltered. DMM epiphyses were significantly expanded from 4-weeks post-surgery compared with healthy baseline and this trend further increased up to 20-weeks (Fig 7B). While at 4-weeks post-surgery we observed a DMM epiphyseal expansion of +19.7% compared with baseline (p = 0.0004, Fig 7B), at 20-weeks post-surgery this increment was up to +38.9% (p<0.0001, 7B). An increase in contralateral epiphyseal volume was also observed from 8-weeks post-surgery (expansion of +33.3% from baseline to 20-weeks, p<0.0001, Fig 7B), which caused a progressive decrease in the difference between DMM-operated/contralateral epiphyseal volume (Fig 7C). Significant expansion of DMM-operated epiphysis was found compared with contralateral at 4-weeks post-surgery (+5.7%, p = 0.025, Fig 7C), and further increased at 8-weeks post-surgery (+9.4%, p = 0.004). However, while at 12-weeks significant expansion (+7.5%, p = 0.035) was still observed, there were no differences in DMM epiphyseal volume at 20-weeks post-surgery (+2.1, p = 0.76).

### Histopathology also revealed early chondrophytes at 1-week post-DMM

Grading of articular cartilage lesions by histological scoring (Fig 6E) revealed OA progression confined to the medial compartment of destabilised joints. Histology (leftmost images in Fig 6B) also revealed very early chondrophytes (non-calcified osteophyte precursors) in the medial margin at 1-week post-DMM and no osteophytes were visible at any time point in contralateral tibiae.

### Structural changes in subchondral were not significantly correlated to the severity of cartilage lesions at individual time points

Structural changes in subchondral bone and osteophyte volume were not significantly correlated to the severity of cartilage lesions at individual time points. However, some clear direct relationships were found between subchondral plate thickness and cartilage score at 4- and 12-weeks post-surgery and between trabecular BV/TV and cartilage score at 1- and 4-weeks (Table 3). Osteophyte volume had a strong linear relationship with articular cartilage score, but only at 20-weeks post-surgery. This can however, be a power issue, since correlations at individual time points were obtained from a relatively small sample size (n = 5). Therefore, we have studied global correlation plots between subchondral bone plate thickness and articular cartilage score, trabecular BV/TV and articular cartilage score, and osteophyte volume and articular cartilage score in the medial aspect of DMM tibiae including all time points post-surgery. Non-parametric correlation analyses have shown significant correlations in the three situations; r’ = 0.78, p<0.0001, for subchondral bone plate thickness *vs*. articular cartilage score, r’ = 0.37, p = 0.04, for trabecular BV/TV *vs*. articular cartilage score and r’ = 0.57, p = 0.006, for osteophyte volume *vs*. articular cartilage score. Consequently, this indicated that severity of lesion progression in subchondral bone, osteophyte formation and articular cartilage was indeed globally associated over time.

**Table 3:** Non-parametric correlations between lesion progression in subchondral bone compartments, osteophyte formation and articular cartilage score.

Time point (weeks)	Subchondral bone plate thickness *vs*. articular cartilage score (Spearman *r’*)	Trabecular BV/TV *vs*. articular cartilage score (Spearman *r’*)	Osteophyte volume *vs*. articular cartilage score (Spearman *r’*)
1	0.44	0.62	
2	0.55	0.20	-0.21
4	0.80	0.70	-0.20
8	-0.40	0.40	0.50
12	0.67	0.21	0.32
20	0.21	0.31	0.82

Subchondral bone plate thickness, trabecular BV/TV, osteophyte volume and articular cartilage score were linearly crossed using non-parametric correlation analysis to determine the association among alterations in subchondral bone microstructure, articular cartilage score and osteophyte formation. Data were analysed in the following pairs: subchondral bone plate thickness *vs*. articular cartilage score, trabecular BV/TV *vs*. articular cartilage score, and osteophyte volume *vs*. articular cartilage score. The non-parametric correlation coefficient (Spearman r’) for each correlation is reported (n = 5)

## Discussion

In this work, we present an automated epiphyseal analysis for characterization of bone changes in experimental OA. Although we confined joint analysis to tibiae, histology has shown that lesions in this model are prevalent in the medial tibial epiphysis [27] and consequently, assessment in tibiae should provide a representative measure of OA changes in the whole joint. On the tibial surface, we generated planar heat maps of subchondral bone thickness to locate the areas of major alterations. From these data, progressive sclerosis was exclusively observed in the medial compartment of operated joints. This supported previous evidence that lesions are time-dependent and confined to load-bearing areas or sites of trauma [38]. Nevertheless, it has been suggested that the cortical plate and trabecular bone have different involvement in OA [39] and should be evaluated separately [40]. Segmentation of cortical and trabecular bone in the tibial epiphysis has been previously performed by manual contouring [16–18], which is time-consuming and subjective. Automated approaches for compartmentalisation proposed in literature rely mostly on thresholds [19–23], a critical parameter in micro-CT segmentation, and are prone to error [15]. For increased robustness, segmentation methods should also consider microstructural criteria, such as thickness differences between cortical and trabecular bone [23, 24]. Our method relies on the degree of macro-porosity, used to classify bone structure at the macroscopic level [32], to distinguish between cortical and trabecular bone and therefore, has the strength of being threshold independent in the differentiation between compartments. We confined volumes-of-interest to the load bearing regions of the tibial plateau where consistent increases in subchondral bone thickness were observed by our planar heat maps. To improve the robustness of the measurements, we applied 3D registration to co-align pairs of DMM/contralateral tibiae prior to analysis. This methodology has demonstrated accurate longitudinal monitoring of bone resorption and apposition [41] and improved sensitivity and reproducibility of micro-architectural quantifications [42, 43]. In our study, it reduced the variability of the measurements, suggesting that the robustness of the method was also improved. Furthermore, we used the paired alignment to determine shape differences between DMM and contralateral tibiae. Shape comparisons have been previously proposed as a potential imaging biomarker of pathology progression in clinical studies [44–46], while in experimental models, and also using alignment by 3D registration, they have demonstrated the ability to track and quantify osteophytes [47]. In our method, we considered that outer shape and size differences between DMM and contralateral tibiae were caused by osteophytes, a valid assumption when comparing pairs of bones from the same animal. Not only did this method demonstrate high accuracy in detecting osteophytes (from 2-weeks post-surgery), but also demonstrated robustness by the strong agreement with manual segmentation and histology. We further proposed epiphyseal volume as a surrogate measurement of osteophyte growth, since osteophytes broaden the articular surface [48]. Early epiphyseal expansion (from 4-weeks post-surgery) was detected by this semi-automated method, thus demonstrating its potential for early osteophyte measurement.

The application of our quantitative methodologies to experimental OA showed very early development of subchondral bone changes and osteophyte formation. These alterations were confined to the medial tibial epiphysis, an observation consistent with the model validation [27] and microstructural studies [16]. Our results support the notion that osteophyte formation might be triggered by load imbalance due to increased laxity [16] and acts to restabilise the joint [48]. This explanation is also supported by clinical evidence in which osteophyte removal increased joint instability [49]. In the subchondral bone compartments, we found plate thickening and increased trabecular bone mass as early as 4-weeks post-operatively compared with contralateral. Furthermore, measurements in the plate were elevated compared with baseline from 2-weeks post-surgery, while trabecular BV/TV showed changes from 1-week post-surgery. While plate thickness continuously increased over time compared with the correspondent contralateral, no changes were detected in trabecular bone after 4-weeks. Mappings showed remodelling of this compartment into compact bone, resulting in almost complete absence of trabeculae at the late stages. We also found changes in the medial compartment of contralateral joints, an observation consistent with Poulet *et al*. [50] using a loading model. These findings emphasise the limitations of using the contralateral joint as a control, a caveat that has also been documented in surgical models [51]. Furthermore, when we measured epiphyseal volume, we also observed significant expansion in contralateral tibiae despite the absence of any osteophytes. Skeletal growth might cause this expansion; increase in bone mass and longitudinal growth are known to occur after mice reach sexual maturity [52] and, in the C57Bl/6 strain, long bone lengthening has been reported to occur until six months of age [53]. Nevertheless, we cannot exclude that the expansion detected represents a change in animal gait as reported by Poulet *et al*. [50]. Indeed, altered biomechanical loading, detected by incapacitance testing, has been reported in the DMM model as a consequence of the painful behaviour developed at the late stages of disease (11-weeks post-surgery) [34]. This seems to be consistent with our study, in which we observed contralateral epiphyseal expansion particularly at later stages of disease. Nevertheless, in the absence of gait and incapacitance data for our cross-sectional study, we can only hypothesize on these findings.

Early subchondral bone changes reported in the mouse include mostly subchondral bone plate thinning, decreased bone mineral density and loss of trabecular bone mass [17, 25]. Additionally, in different animal models, including the mouse, thickening has been described as a late event in which sclerosis is preceded by resorption [54, 55], but we were not able to replicate these findings. This might be due to the nature of the DMM model that induces very mild, slow-progressing lesions which might are not associated to significant inflammation but alter the mechanical loading of the joint [51, 56]. This would explain why the bone response appear consistent with bone overloading only without an inflammatory, resorption-inducing phase. Alternatively, this might be due to insufficient sensitivity of micro-CT imaging compared to histomorphometry in detecting transient, minor changes in bone turnover that might be significant at cellular level, but not as clear in terms of bone mass.

Our results demonstrate that, using highly sensitive 3D imaging, structural changes in subchondral bone and osteophyte formation can be detected from 2-weeks post-surgery compared with healthy mice and 4-weeks post-surgery compared with contralateral, both time points where conventional articular cartilage degradation scoring (achieved by histology) was unable to detect significant pathological changes. The temporal progression of articular cartilage score in the current study is, however, consistent with the temporal progression of structural changes in hyaline cartilage assessed in 3D by contrast enhanced micro-CT in our previous study, where significant structural changes in articular cartilage thickness where observed from 4-weeks post-surgery [29]. Our results suggest a temporal sequence of detectable structural changes in bone occurring before measurable degradation (by conventional histopathology scoring) in cartilage, but, in no way, they implicate that cell activation and macro/nano-scale tissue remodelling in the two tissues follow the same temporal order. Nonetheless, the present study indicates that early alterations in subchondral bone are quantifiable and could be used to monitor progression of disease in *in vivo* studies.

## Conclusions

The methods described in the current report provide a robust, high-throughput, platform for the quantitative assessment of epiphyseal bone changes in experimental OA. In our cross-sectional study, we demonstrated that progressive structural changes in subchondral bone and osteophyte formation are detectable as early as 4-weeks after DMM surgery, despite articular cartilage score did not show significant changes until 12 weeks post-DMM surgery. Our automated quantitative imaging methods will allow to sensitively and rapidly measure disease progression on periarticular bone in a range of experimental models of murine OA, with the possibility of extending its use to longitudinal studies using *in vivo* micro-CT scanners.

## Supporting information

S1 Table Coefficient of variation (%) of the measurements in subchondral bone compartments with and without pre-alignment by 3D image registration. (DOCX)

S1 Dataset Dataset underlying the findings reported in the manuscript. (XLSX)

## References

[1] Lories RJ, Luyten FP. The bone-cartilage unit in osteoarthritis. Nat Rev Rheumatol. 2011;7(1):43–9. 10.1038/nrrheum.2010.197 21135881

[2] Weinans H. Periarticular bone changes in osteoarthritis. HSS J. 2012;8(1):10–2. PubMed Central PMCID: PMCPMC3295953. 10.1007/s11420-011-9257-5 22423223 PMC3295953

[3] Goldring SR. Alterations in periarticular bone and cross talk between subchondral bone and articular cartilage in osteoarthritis. Ther Adv Musculoskelet Dis. 2012;4(4):249–58. PubMed Central PMCID: PMCPMC3403248. 10.1177/1759720X12437353 22859924 PMC3403248

[4] Koh YH, Hong SH, Kang HS, Chung CY, Koo KH, Chung HW, et al The effects of bone turnover rate on subchondral trabecular bone structure and cartilage damage in the osteoarthritis rat model. Rheumatol Int. 2010;30(9):1165–71. 10.1007/s00296-009-1118-x 19711077

[5] Maas O, Joseph GB, Sommer G, Wild D, Kretzschmar M. Association between cartilage degeneration and subchondral bone remodeling in patients with knee osteoarthritis comparing MRI and (99m)Tc-DPD-SPECT/CT. Osteoarthritis Cartilage. 2015;23(10):1713–20. 10.1016/j.joca.2015.05.014 26028141

[6] Kaukinen P, Podlipska J, Guermazi A, Niinimaki J, Lehenkari P, Roemer FW, et al Associations between MRI-defined structural pathology and generalized and localized knee pain—the Oulu Knee Osteoarthritis study. Osteoarthritis Cartilage. 2016;24(9):1565–76. 10.1016/j.joca.2016.05.001 27174007

[7] Bolbos RI, Zuo J, Banerjee S, Link TM, Ma CB, Li X, et al Relationship between trabecular bone structure and articular cartilage morphology and relaxation times in early OA of the knee joint using parallel MRI at 3 T. Osteoarthritis Cartilage. 2008;16(10):1150–9. PubMed Central PMCID: PMCPMC2580796. 10.1016/j.joca.2008.02.018 18387828 PMC2580796

[8] Kwan Tat S, Lajeunesse D, Pelletier JP, Martel-Pelletier J. Targeting subchondral bone for treating osteoarthritis: what is the evidence? Best Pract Res Clin Rheumatol. 2010;24(1):51–70. PubMed Central PMCID: PMCPMC5250505. 10.1016/j.berh.2009.08.004 20129200 PMC5250505

[9] Barr AJ, Campbell TM, Hopkinson D, Kingsbury SR, Bowes MA, Conaghan PG. A systematic review of the relationship between subchondral bone features, pain and structural pathology in peripheral joint osteoarthritis. Arthritis Res Ther. 2015;17(1):228. PubMed Central PMCID: PMCPMC4548899. 26303219 10.1186/s13075-015-0735-x PMC4548899

[10] Vincent TL, Williams RO, Maciewicz R, Silman A, Garside P, Arthritis Research UKamwg. Mapping pathogenesis of arthritis through small animal models. Rheumatology (Oxford). 2012;51(11):1931–41. 22427408 10.1093/rheumatology/kes035

[11] Roemer FW, Eckstein F, Hayashi D, Guermazi A. The role of imaging in osteoarthritis. Best Pract Res Clin Rheumatol. 2014;28(1):31–60. 10.1016/j.berh.2014.02.002 24792944

[12] Felson DT, Gale DR, Elon Gale M, Niu J, Hunter DJ, Goggins J, et al Osteophytes and progression of knee osteoarthritis. Rheumatology (Oxford). 2005;44(1):100–4. 15381791 10.1093/rheumatology/keh411

[13] Kellgren JH, Lawrence JS. Radiological assessment of osteo-arthrosis. Ann Rheum Dis. 1957;16(4):494–502. PubMed Central PMCID: PMCPMC1006995. 13498604 10.1136/ard.16.4.494 PMC1006995

[14] Guermazi A, Hayashi D, Roemer FW, Felson DT. Osteoarthritis: a review of strengths and weaknesses of different imaging options. Rheum Dis Clin North Am. 2013;39(3):567–91. 10.1016/j.rdc.2013.02.001 23719076

[15] Bouxsein ML, Boyd SK, Christiansen BA, Guldberg RE, Jepsen KJ, Muller R. Guidelines for assessment of bone microstructure in rodents using micro-computed tomography. J Bone Miner Res. 2010;25(7):1468–86. 10.1002/jbmr.141 20533309

[16] Moodie JP, Stok KS, Muller R, Vincent TL, Shefelbine SJ. Multimodal imaging demonstrates concomitant changes in bone and cartilage after destabilisation of the medial meniscus and increased joint laxity. Osteoarthritis Cartilage. 2011;19(2):163–70. 10.1016/j.joca.2010.11.006 21094262

[17] Christiansen BA, Anderson MJ, Lee CA, Williams JC, Yik JH, Haudenschild DR. Musculoskeletal changes following non-invasive knee injury using a novel mouse model of post-traumatic osteoarthritis. Osteoarthritis Cartilage. 2012;20(7):773–82. 10.1016/j.joca.2012.04.014 22531459

[18] Khorasani MS, Diko S, Hsia AW, Anderson MJ, Genetos DC, Haudenschild DR, et al Effect of alendronate on post-traumatic osteoarthritis induced by anterior cruciate ligament rupture in mice. Arthritis Res Ther. 2015;17:30 PubMed Central PMCID: PMCPMC4355375. 10.1186/s13075-015-0546-0 25888819 PMC4355375

[19] Buie HR, Campbell GM, Klinck RJ, MacNeil JA, Boyd SK. Automatic segmentation of cortical and trabecular compartments based on a dual threshold technique for in vivo micro-CT bone analysis. Bone. 2007;41(4):505–15. 10.1016/j.bone.2007.07.007 17693147

[20] Botter S, Van Osch G, Waarsing J, Day J, Verhaar J, Pols H, et al Quantification of subchondral bone changes in a murine osteoarthritis model using micro-CT. Biorheology. 2006;43(3, 4):379–88. 16912410

[21] Burghardt AJ, Buie HR, Laib A, Majumdar S, Boyd SK. Reproducibility of direct quantitative measures of cortical bone microarchitecture of the distal radius and tibia by HR-pQCT. Bone. 2010;47(3):519–28. PubMed Central PMCID: PMCPMC2926164. 10.1016/j.bone.2010.05.034 20561906 PMC2926164

[22] Ding M, Odgaard A, Hvid I. Accuracy of cancellous bone volume fraction measured by micro-CT scanning. J Biomech. 1999;32(3):323–6. 10093033 10.1016/s0021-9290(98)00176-6

[23] Besler BA, Sondergaard RE, Muller R, Stok KS. Reproducibility of compartmental subchondral bone morphometry in the mouse tibiofemoral joint. Bone. 2015;81:649–53. 10.1016/j.bone.2015.09.014 26424216

[24] Lublinsky S, Ozcivici E, Judex S. An automated algorithm to detect the trabecular-cortical bone interface in micro-computed tomographic images. Calcif Tissue Int. 2007;81(4):285–93. 10.1007/s00223-007-9063-8 17828460

[25] Botter SM, van Osch GJ, Clockaerts S, Waarsing JH, Weinans H, van Leeuwen JP. Osteoarthritis induction leads to early and temporal subchondral plate porosity in the tibial plateau of mice: an in vivo microfocal computed tomography study. Arthritis Rheum. 2011;63(9):2690–9. 10.1002/art.30307 21360519

[26] Siebelt M, Waarsing JH, Groen HC, Muller C, Koelewijn SJ, de Blois E, et al Inhibited osteoclastic bone resorption through alendronate treatment in rats reduces severe osteoarthritis progression. Bone. 2014;66:163–70. 10.1016/j.bone.2014.06.009 24933343

[27] Glasson SS, Blanchet TJ, Morris EA. The surgical destabilization of the medial meniscus (DMM) model of osteoarthritis in the 129/SvEv mouse. Osteoarthritis Cartilage. 2007;15(9):1061–9. 10.1016/j.joca.2007.03.006 17470400

[28] Doube M, Klosowski MM, Arganda-Carreras I, Cordelieres FP, Dougherty RP, Jackson JS, et al BoneJ: Free and extensible bone image analysis in ImageJ. Bone. 2010;47(6):1076–9. PubMed Central PMCID: PMCPMC3193171. 10.1016/j.bone.2010.08.023 20817052 PMC3193171

[29] Das Neves Borges P, Forte AE, Vincent TL, Dini D, Marenzana M. Rapid, automated imaging of mouse articular cartilage by microCT for early detection of osteoarthritis and finite element modelling of joint mechanics. Osteoarthritis Cartilage. 2014;22(10):1419–28. PubMed Central PMCID: PMCPMC4192140. 10.1016/j.joca.2014.07.014 25278053 PMC4192140

[30] Otsu N. A threshold selection method from gray-level histograms. Automatica. 1975;11(285–296):23–7.

[31] Studholme C, Hill DLG, Hawkes DJ. An overlap invariant entropy measure of 3D medical image alignment. Pattern Recognition. 1999;32(1):71–86.

[32] Rho JY, Kuhn-Spearing L, Zioupos P. Mechanical properties and the hierarchical structure of bone. Med Eng Phys. 1998;20(2):92–102. PubMed Central PMCID: PMC9679227. 9679227 10.1016/s1350-4533(98)00007-1

[33] Chia SL, Sawaji Y, Burleigh A, McLean C, Inglis J, Saklatvala J, et al Fibroblast growth factor 2 is an intrinsic chondroprotective agent that suppresses ADAMTS‐5 and delays cartilage degradation in murine osteoarthritis. Arthritis & Rheumatism. 2009;60(7):2019–27. 19565481 10.1002/art.24654

[34] Inglis JJ, McNamee KE, Chia SL, Essex D, Feldmann M, Williams RO, et al Regulation of pain sensitivity in experimental osteoarthritis by the endogenous peripheral opioid system. Arthritis Rheum. 2008;58(10):3110–9. 10.1002/art.23870 18821665

[35] Glasson SS, Askew R, Sheppard B, Carito B, Blanchet T, Ma HL, et al Deletion of active ADAMTS5 prevents cartilage degradation in a murine model of osteoarthritis. Nature. 2005;434(7033):644–8. 10.1038/nature03369 15800624

[36] Altman DG, Bland JM. Measurement in Medicine: The Analysis of Method Comparison Studies. The Statistician. 1983;32(3):307.

[37] Giavarina D. Understanding Bland Altman analysis. Biochem Med (Zagreb). 2015;25(2):141–51. PubMed Central PMCID: PMCPMC4470095. 26110027 10.11613/BM.2015.015 PMC4470095

[38] Iijima H, Aoyama T, Tajino J, Ito A, Nagai M, Yamaguchi S, et al Subchondral plate porosity colocalizes with the point of mechanical load during ambulation in a rat knee model of post-traumatic osteoarthritis. Osteoarthritis Cartilage. 2016;24(2):354–63. 10.1016/j.joca.2015.09.001 26376125

[39] Burr DB, Gallant MA. Bone remodelling in osteoarthritis. Nat Rev Rheumatol. 2012;8(11):665–73. 10.1038/nrrheum.2012.130 22868925

[40] Goldring SR, Goldring MB. Bone and cartilage in osteoarthritis: is what's best for one good or bad for the other? Arthritis Res Ther. 2010;12(5):143 PubMed Central PMCID: PMCPMC2991002. 10.1186/ar3135 21044355 PMC2991002

[41] Lambers FM, Schulte FA, Kuhn G, Webster DJ, Muller R. Mouse tail vertebrae adapt to cyclic mechanical loading by increasing bone formation rate and decreasing bone resorption rate as shown by time-lapsed in vivo imaging of dynamic bone morphometry. Bone. 2011;49(6):1340–50. 10.1016/j.bone.2011.08.035 21964411

[42] Nishiyama KK, Campbell GM, Klinck RJ, Boyd SK. Reproducibility of bone micro-architecture measurements in rodents by in vivo micro-computed tomography is maximized with three-dimensional image registration. Bone. 2010;46(1):155–61. 10.1016/j.bone.2009.09.023 19796719

[43] Campbell GM, Tiwari S, Grundmann F, Purcz N, Schem C, Gluer CC. Three-dimensional image registration improves the long-term precision of in vivo micro-computed tomographic measurements in anabolic and catabolic mouse models. Calcif Tissue Int. 2014;94(3):282–92. 10.1007/s00223-013-9809-4 24170302

[44] Gregory JS, Waarsing JH, Day J, Pols HA, Reijman M, Weinans H, et al Early identification of radiographic osteoarthritis of the hip using an active shape model to quantify changes in bone morphometric features: can hip shape tell us anything about the progression of osteoarthritis? Arthritis Rheum. 2007;56(11):3634–43. 10.1002/art.22982 17968890

[45] Merle C, Waldstein W, Gregory JS, Goodyear SR, Aspden RM, Aldinger PR, et al How many different types of femora are there in primary hip osteoarthritis? An active shape modeling study. J Orthop Res. 2014;32(3):413–22. 10.1002/jor.22518 24249665

[46] Barr RJ, Gregory JS, Reid DM, Aspden RM, Yoshida K, Hosie G, et al Predicting OA progression to total hip replacement: can we do better than risk factors alone using active shape modelling as an imaging biomarker? Rheumatology (Oxford). 2012;51(3):562–70. 22139532 10.1093/rheumatology/ker382

[47] Saha PK, Liang G, Elkins JM, Coimbra A, Duong LT, Williams DS, et al A new osteophyte segmentation algorithm using partial shape model and its applications to rabbit femur anterior cruciate ligament transection via micro-CT imaging. IEEE Trans Biomed Eng. 2011;58(8):2212–27. PubMed Central PMCID: PMCPMC4910393. 10.1109/TBME.2011.2129519 PMC4910393 21421428

[48] van der Kraan PM, van den Berg WB. Osteophytes: relevance and biology. Osteoarthritis Cartilage. 2007;15(3):237–44. 10.1016/j.joca.2006.11.006 17204437

[49] Pottenger LA, Phillips FM, Draganich LF. The effect of marginal osteophytes on reduction of varus-valgus instability in osteoarthritic knees. Arthritis Rheum. 1990;33(6):853–8. 2363739 10.1002/art.1780330612

[50] Poulet B, de Souza R, Kent AV, Saxon L, Barker O, Wilson A, et al Intermittent applied mechanical loading induces subchondral bone thickening that may be intensified locally by contiguous articular cartilage lesions. Osteoarthritis Cartilage. 2015;23(6):940–8. PubMed Central PMCID: PMCPMC4459965. 10.1016/j.joca.2015.01.012 25655679 PMC4459965

[51] Fang H, Beier F. Mouse models of osteoarthritis: modelling risk factors and assessing outcomes. Nat Rev Rheumatol. 2014;10(7):413–21. 10.1038/nrrheum.2014.46 24662645

[52] Jilka RL. The relevance of mouse models for investigating age-related bone loss in humans. J Gerontol A Biol Sci Med Sci. 2013;68(10):1209–17. PubMed Central PMCID: PMCPMC3779631. 10.1093/gerona/glt046 23689830 PMC3779631

[53] Glatt V, Canalis E, Stadmeyer L, Bouxsein ML. Age-related changes in trabecular architecture differ in female and male C57BL/6J mice. J Bone Miner Res. 2007;22(8):1197–207. 10.1359/jbmr.070507 17488199

[54] Anderson-MacKenzie JM, Quasnichka HL, Starr RL, Lewis EJ, Billingham ME, Bailey AJ. Fundamental subchondral bone changes in spontaneous knee osteoarthritis. Int J Biochem Cell Biol. 2005;37(1):224–36. 10.1016/j.biocel.2004.06.016 15381164

[55] Intema F, Hazewinkel HA, Gouwens D, Bijlsma JW, Weinans H, Lafeber FP, et al In early OA, thinning of the subchondral plate is directly related to cartilage damage: results from a canine ACLT-meniscectomy model. Osteoarthritis Cartilage. 2010;18(5):691–8. 10.1016/j.joca.2010.01.004 20175978

[56] Little CB, Smith MM. Animal models of osteoarthritis. Current Rheumatology Reviews. 2008;4(3):175–82.

