# Supplementary material for: Automated assessment of bone changes in cross-sectional micro-CT studies of murine experimental osteoarthritis
Source: PLoS One. 2017 Mar 23;12(3):e0174294. doi: 10.1371/journal.pone.0174294 (PMC5363908; doi:10.1371/journal.pone.0174294)
Supplement: S1 Table — (DOCX) [file pone.0174294.s001.docx]

| Microstructural parameter | | | **Coefficient of Variation (%)** | |
| --- | --- | --- | --- | --- |
|  |  |  | With pre-alignment | Without pre-alignment |
| Subchondral bone plate volume (mm^3^) | Medial | DMM | 21.3 | 22.4 |
|  |  | Contralateral | 19.6 | 21.0 |
|  | Lateral | DMM | 10.8 | 8.1 |
|  |  | Contralateral | 13.0 | 10.6 |
| Subchondral bone plate thickness (µm) | Medial | DMM | 21.0 | 22.9 |
|  |  | Contralateral | 19.4 | 21.4 |
|  | Lateral | DMM | 9.8 | 8.4 |
|  |  | Contralateral | 11.2 | 11.1 |
| Trabecular total volume (mm^3^) | Medial | DMM | 42.4 | 51.9 |
|  |  | Contralateral | 23.4 | 25.6 |
|  | Lateral | DMM | 4.3 | 3.9 |
|  |  | Contralateral | 5.3 | 5.5 |
| Trabecular bone volume (mm^3^) | Medial | DMM | 37.2 | 48.2 |
|  |  | Contralateral | 17.5 | 20.9 |
|  | Lateral | DMM | 5.1 | 8.1 |
|  |  | Contralateral | 5.3 | 8.0 |
| Trabecular BV/TV (%) | Medial | DMM | 8.3 | 8.5 |
|  |  | Contralateral | 9.7 | 8.4 |
|  | Lateral | DMM | 6.9 | 8.2 |
|  |  | Contralateral | 7.0 | 8.3 |

Coefficient of variation (%CV) in subchondral bone plate volume, thickness, and trabecular total volume, bone volume, and BV/TV measured with and without pre-alignment by 3D image registration were paired and analysed. Data were divided in sub-groups, including the medial and lateral aspects of the tibial plateau in DMM and contralateral, the %CV reported was obtained by averaging the %CV of the measurements in all six time points post-surgery.
